# Protein translation: biological processes and therapeutic strategies for human diseases

**DOI:** 10.1038/s41392-024-01749-9

**Published:** 2024-02-23

**Authors:** Xuechao Jia, Xinyu He, Chuntian Huang, Jian Li, Zigang Dong, Kangdong Liu

**Affiliations:** 1 Department of Pathophysiology, School of Basic Medical Sciences, Academy of Medical Sciences, Zhengzhou University, Zhengzhou, Henan 450000 China; 2 China-US (Henan) Hormel Cancer Institute, Zhengzhou, Henan 450000 China; 3 Department of Pathology and Pathophysiology, Henan University of Chinese Medicine, Zhengzhou, Henan 450000 China; 4 Tianjian Laboratory of Advanced Biomedical Sciences, Zhengzhou, Henan 450052 China; 5 Research Center for Basic Medicine Sciences, Academy of Medical Sciences, Zhengzhou University, Zhengzhou, 450052 Henan China; 6 Provincial Cooperative Innovation Center for Cancer Chemoprevention, Zhengzhou University, Zhengzhou, Henan 450000 China; 7 State Key Laboratory of Esophageal Cancer Prevention and Treatment, Zhengzhou University, Zhengzhou, Henan 450000 China; 8 The Collaborative Innovation Center of Henan Province for Cancer Chemoprevention, Zhengzhou, Henan 450000 China

**Keywords:** Oncogenes, Tumour biomarkers

## Abstract

Protein translation is a tightly regulated cellular process that is essential for gene expression and protein synthesis. The deregulation of this process is increasingly recognized as a critical factor in the pathogenesis of various human diseases. In this review, we discuss how deregulated translation can lead to aberrant protein synthesis, altered cellular functions, and disease progression. We explore the key mechanisms contributing to the deregulation of protein translation, including functional alterations in translation factors, tRNA, mRNA, and ribosome function. Deregulated translation leads to abnormal protein expression, disrupted cellular signaling, and perturbed cellular functions- all of which contribute to disease pathogenesis. The development of ribosome profiling techniques along with mass spectrometry-based proteomics, mRNA sequencing and single-cell approaches have opened new avenues for detecting diseases related to translation errors. Importantly, we highlight recent advances in therapies targeting translation-related disorders and their potential applications in neurodegenerative diseases, cancer, infectious diseases, and cardiovascular diseases. Moreover, the growing interest lies in targeted therapies aimed at restoring precise control over translation in diseased cells is discussed. In conclusion, this comprehensive review underscores the critical role of protein translation in disease and its potential as a therapeutic target. Advancements in understanding the molecular mechanisms of protein translation deregulation, coupled with the development of targeted therapies, offer promising avenues for improving disease outcomes in various human diseases. Additionally, it will unlock doors to the possibility of precision medicine by offering personalized therapies and a deeper understanding of the molecular underpinnings of diseases in the future.

## Introduction

**Fig. 1:**
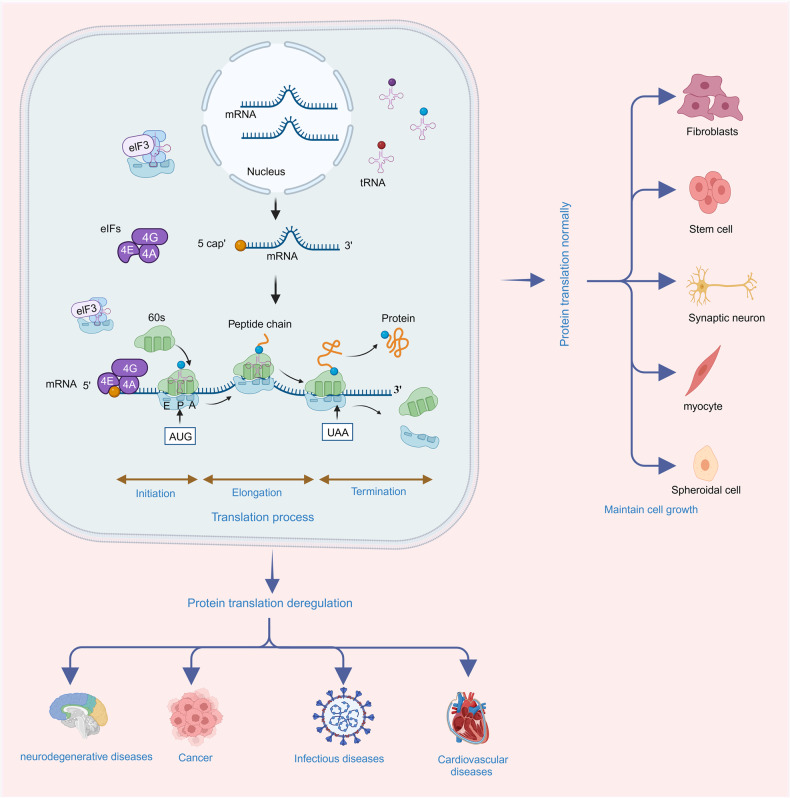
Protein translation deregulation and its related human disease. Protein translation includes three processes of initiation, elongation and termination. With the participation of ribosome, mRNA, tRNA, and translation related factors, the protein translation process enrolls in orderly to synthesis the nascent peptides accurately, thus, maintaining the cell proliferation and differentiation accurately. When this process is deregulated, the abundance, stability or functions of translated peptides alter and the cell fates run into disease states. Protein translation deregulation leads to neurodegenerative diseases, cancer, infectious diseases, cardiovascular diseases and other diseases

Protein translation, also known as protein synthesis, is a fundamental biological process that involves the conversion of the nucleotide sequence in mRNA into a specific sequence of amino acids, forming a functional protein. This process is essential for all living organisms and plays a central role in gene expression, allowing cells to produce the proteins necessary for their structure, function, and regulation. Protein translation consists of several key steps, including initiation, elongation, and termination (Fig. 1). Initiation typically involves the small ribosomal subunit binding to the mRNA, guided by initiation factors, and scanning for the start codon (usually AUG). Once the start codon is recognized, the large ribosomal subunit joins, and protein synthesis begins with transfer RNA (tRNA) molecules bringing in amino acids to build the growing polypeptide chain. During elongation, tRNA molecules bring in amino acids that match the codons on mRNA. The ribosome moves in a 5’ to 3’ direction along the mRNA, and the tRNA that was previously in the A (aminoacyl) site is moved to the P (peptidyl) site and then the E (exit) site. This allows the next codon on mRNA to enter the A site continuing elongation and peptide bond formation. During termination, release factors (RFs) recognize the stop codon and bind to the A site of the ribosome. This binding triggers the hydrolysis of the bond between the completed polypeptide chain and the final tRNA in the P site, resulting in release of polypeptide from the ribosome.

**Fig. 2:**
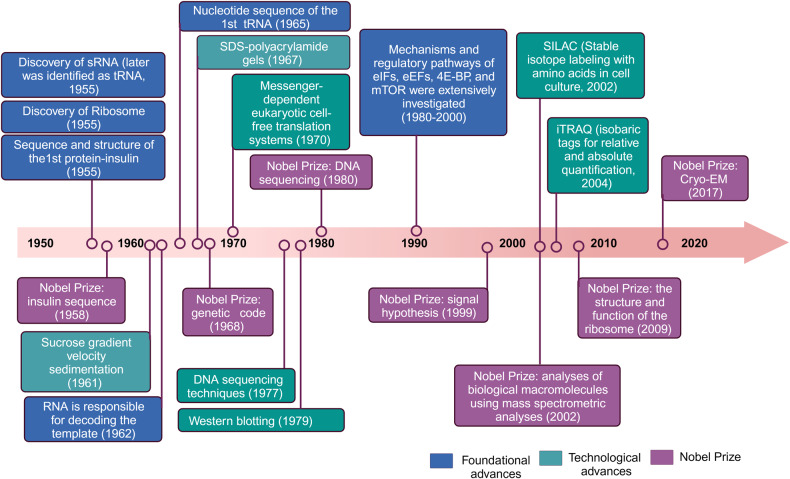
Timeline of discoveries and Nobel Prize in the fields of protein translation

Researches focusing on protein translation has been studied over the last decades (Fig. 2).^1^ Before the 1950s, research addressing physiological questions of protein translation was largely descriptive in nature.^2^ In the 1950s, the discovery of tRNA and the characterization of ribosome laid the foundation for understanding the intricate molecular machinery involved in translation.^3–5^ In 1955, Sanger determined the sequence and structure of the first protein (bovine insulin), earning him the Nobel Prize in chemistry three years later.^6^ Advancements in molecular biology and biochemistry during the 1960s–1970s, led to the elucidation of the genetic code, translation factors and other components of the eukaryotic translation system.^7,8^ For instance, in 1962, chemical modifications of amino acids substantiated that the RNA component was responsible for decoding the template.^9^ Subsequently, in 1965, the complete nucleotide sequence of the first nucleic acid, alanine transfer RNA, was determined.^10^ Three years later, this achievement led to the recognition for the interpretation of the genetic code and its function in protein synthesis.^11^ Several technological and foundational advancements emerged during this time frame, including Sucrose gradient velocity sedimentation (1961),^12^ SDS-polyacrylamide gels (1967),^13^ Western blotting (1979),^14^ and messenger-dependent eukaryotic cell-free translation systems (1970),^15^ which researchers began to extensively utilize. Furthermore, the successful application of DNA sequencing techniques in 1977 led to its recognition with the Nobel Prize in 1980.^16^ During the 1980s–1990s, appreciation for mechanistic and regulatory pathways expands rapidly.^17^ For example, detailed investigations into the signal pathways involving translation initiation factors, elongation factors, 4E-BP, and mTOR were conducted extensively during this period.^1^ Moreover, the signal hypothesis, proposing that proteins possess intrinsic signals governing their transport and localization within the cell, was discovered and subsequently awarded the Nobel Prize in Physiology or Medicine in 1999.^18^ Since then, significant progress has been made in structural biology with the determination of high-resolution structures of ribosomes and translation-related complexes that shed light on the detailed mechanisms of translation.^19,20^ The Nobel Prize was awarded in 2002 for the identification and structure analyses of biological macromolecules using mass spectrometric analyses, in 2009 for studies of the structure and function of the ribosome, and in 2017 for the development of cryo-electron microscopy, allowing high-resolution structure determination of biomolecules in solution. Furthermore, the advent of genomics and proteomics has enabled researchers to explore translation on a global scale, uncovering the complexity of translation regulation and its role in various cellular processes and diseases.^21–23^

Protein translation deregulation refers to the disruption or alteration of the normal process of protein synthesis, resulting in aberrant protein production or impaired regulation of protein expression.^24,25^ It encompasses various mechanisms that can affect different stages of translation. In this process, deregulation can occur at the expression level through mutation or modification of mRNA, tRNA, translation factors, ribosomes, or regulatory elements.^25–27^ These deregulations can impair fidelity of translation and increase the occurrence of translation errors such as incorrect amino acid incorporation or premature termination, leading to the synthesis of defective or non-functional proteins and deregulated protein localization.^27^ Translation deregulation can be induced by various factors including epigenetic modifications, genetic mutations, deregulated translation factors or regulatory proteins, alterations in mRNA stability or localization, as well as environmental and cellular stress conditions.^28–30^

Protein translation deregulation, with its various underlying mechanisms affecting translation, has far-reaching consequences in human diseases, such as neurodegenerative diseases, cancer, infectious diseases, cardiovascular diseases (CVDs) (Fig. 1).^31–36^ The dysregulation can alter protein expression, abnormal protein isoforms, or impaired protein quality control. The dysregulated expression of specific proteins can disrupt normal cellular processes, signaling pathways, and molecular networks, contributing to disease pathogenesis.^37^ Deregulation of protein translation can result in the production of aberrant protein isoforms with altered sequences, truncations or modifications.^33^ These abnormal isoforms may have altered functions, loss of regulatory control or gain of toxic properties. When protein translation is dysregulated, the load of misfolded or aberrant proteins may overwhelm the cellular quality control machinery leading to the accumulation of toxic protein aggregates and proteinopathies.^38^ Overall, deregulation of protein translation plays a significant role in human disease by influencing protein expression, function, and cellular homeostasis. Elucidating the mechanisms of protein translation deregulation in specific diseases can provide insights into disease pathogenesis and guide the development of novel therapeutic strategies.

## Mechanisms of protein translation deregulation

### Regulation of translation initiation

**Fig. 3:**
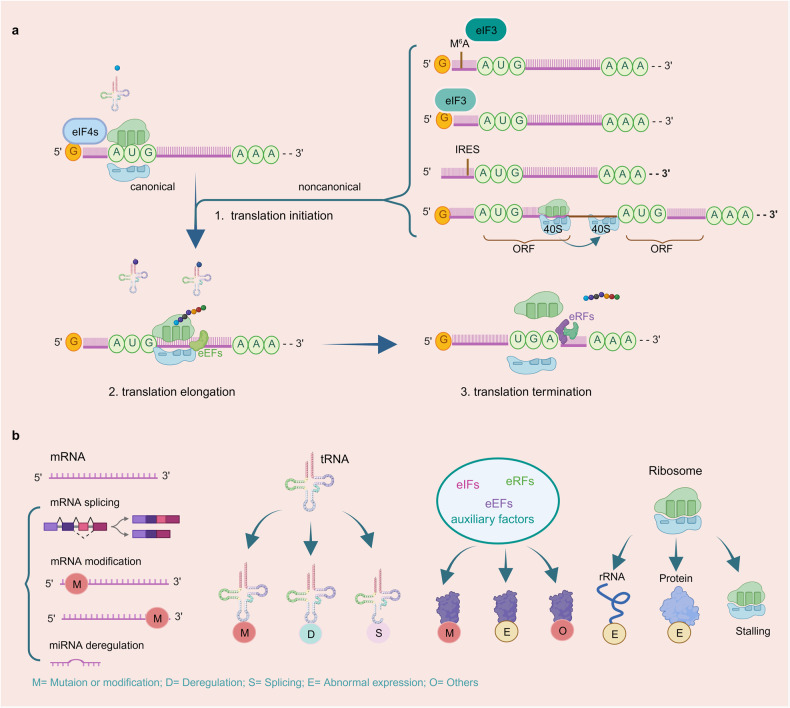
The mechanism of protein translation process and protein translation deregulation manners. **a** Process of protein translation. 1. Translation initiation: The canonical translation initiation starts with recognizing and binding with 5′-cap domain of the mRNA by eIF4E complex in a cap dependent manner. After binding with cap, the mRNA translation is activated and recruits the 43 S ribosomal subunit to the 5’ end of the mRNA. Upon the 43 S ribosomal subunit scanning and recognizing the AUG start codon in the mRNA, 60 S ribosomal subunit is recruited and forms an 80 S ribosome complex to contribute for peptides elongation. noncanonical translation initiation vary in the aspects of eIFs categories, the cap recognition manner and the other conditions. The currently known noncanonical translation initiation includes m^6^A translation initiation, eIF3d translation initiation, IRESs-mediated translation initiation and ribosome shunting. 2. Translation elongation: In this process, the ribosome moves along the mRNA in the 5’ to 3’ direction with the attending of eEFs, the aminoacyl-tRNA in the A site forms a peptide bond with the growing polypeptide chain attached to the tRNA in the P site. The uncharged tRNA shifts from the P site to the E site and the peptidyl-tRNA from the A site to the P site. Then the uncharged tRNA in the E site is released from the ribosome, making way for the next aminoacyl-tRNA to enter the A site and repeat the process. 3. Translation termination: when ribosome complex recognizes a stop codon, termination is triggered. This process is mediated by the release factors eRF1 and eRF3. eRF1 regulates the nascent polypeptide release from the P-site peptidyl-tRNA, whereas eRF3 enhances polypeptide release. **b** Protein translation deregulation mechanism in mRNA, tRNA, translation factors and ribosome. mRNA: alternative splicing, mutation or modification in 5’ or 3’ UTR of mRNA. tRNA: mutation or modification of tRNA, tRNA deregulation and abnormal splicing. Translational factors: mutation, modification, abnormal expression and other variations. Ribosome: mutation or abnormal expression of ribosome components, ribosome stalling and so on

Translation initiation is a highly regulated process that governs the initiation of protein synthesis in cells. It involves the assembly of the translation machinery at the start codon of mRNA, typically AUG (methionine) in eukaryotes. Depending on the initiation model, translation initiation can be classified into canonical and noncanonical modes (Fig. 3a). The canonical translation initiation process commences with the recognition and subsequent binding of the 5’-cap (m7GpppN) domain of mRNA by the eukaryotic initiation factor (eIF)4E complex (comprising eIF4E, eIF4G, and eIF4A) in a cap-dependent manner. Upon binding to the cap, eIF4E recruits the 43 S ribosomal subunit to the 5’ end of the mRNA, thus activating mRNA translation. This subunit is formed through the interaction of an eIF2•Met-tRNAi•GTP ternary complex and the 40 S ribosome complex. After recruitment, the 43 S ribosomal subunit scans along the mRNA in a 5’ to 3’ direction until it recognizes the AUG start codon. Subsequently, joining of the 60 S ribosomal subunit forms an elongation-competent ribosome (80 S) for peptide elongation contribution.^17,39–41^ While mechanisms underlying noncanonical translation initiation vary in terms categories involving different eIFs, they share common features such as cap recognition and ribosome scanning manner as well as other conditions. Noncanonical translation initiations currently known include N(6)-methyl adenosine (m^6^A) translation initiation, internal ribosome entry sites (IRESs)-mediated translation initiation, eIF3d translation initiation, and ribosome shunting initiation (Fig. 3a).^42^ m^6^A modification is commonly found in both 3’ UTR and 5’ UTR regions of eukaryotic mRNAs.^43^ In the 5’ UTR, m^6^A modification can facilitate translation independently of 5’ cap-binding proteins, particularly in response to cellular stress.^44^ Specifically, a single m^6^A modification in the 5’ UTR directly interacts with eIF3, thereby independently recruiting 43 S complex for translation initiation even in the absence of the cap-binding factor eIF4E. Selectively inhibition of adenosine methylation reduces translation efficiency of mRNAs harboring m^6^A in their 5’ UTRs. For example, elevated levels of m^6^A in the Hsp70 mRNA control its cap-independent translation when cells experience heat shock.^45^ In the eIF3d mediated translation initiation, eIF3d, a subunit of the eIF3 complex, has a cap-binding activity, which allows it to recognize the mRNA cap structure. eIF3d can operate cap-dependent translation initiation pathway independently of eIF4E, which was previously considered essential for cap recognition.^46,47^ IRES-mediated translation initiation refers to a mechanism by which certain mRNA molecules, often found in viruses, can initiate protein synthesis within an eukaryotic cell without relying on the traditional cap-dependent translation initiation.^48–50^ IRESs can interact directly with the ribosome and other initiation factors, allowing the ribosome to directly access the start codon without the need for scanning from the 5’ cap. This enables the rapid initiation of protein synthesis, which is crucial for viruses to hijack cellular translation machinery to produce their own proteins within host cells. Ribosome shunting initiation is often observed in plant viruses.^51,52^ Unlike the typical scanning mechanism where ribosomes start translation at the 5’ cap and move along the mRNA in a linear fashion until they find the start codon, ribosome shunting allows the 40 S to bypass certain sections of mRNA and directly jump or “shunt” to a specific downstream start codon, promoting the initiation of protein translation process.

According to the specific combination and interplay manner, these regulatory mechanisms can vary depending on the cellular stress, developmental stage, viral infections and other conditions.^51,53,54^ During translational initiation process in cells, mRNA serves as a template, tRNA functions as an amino acid transporter, ribosomal rRNA provides the translation sites and eukaryotic initiation factors, along with other auxiliary proteins work collaboratively to tightly regulate protein synthesis initiation and ensure precise control of gene expression.^33^ Protein expression dysregulation and post-translational modifications and mutations of initiation-related proteins frequently lead to the inhibition of the translation initiation process.^55–57^ Deregulation of tRNA, including altered tRNA expression, tRNA modifications, tRNA aminoacylation defects, tRNA splicing and maturation defects can contribute to cellular dysfunction and disease.^58–60^ Abnormal expression or mutations of rRNA and ribosome proteins in the ribosome cause aberrant ribosome biogenesis, impairing ribosome functions and inhibiting the translation process.^61,62^ Deregulations in ribosome such as RPS19, RPS14 and others can lead to a spectrum of diseases including Diamond–Black fan anemia, myelodysplastic syndrome and bone and skeletal abnormalities.^36,63,64^ In eukaryotes, the presence of a 5’ cap structure on mRNA is crucial for translation initiation. Modifications in 5’ and 3’ UTRs as well as deregulation of enzymes involved in cap formation or cap-binding proteins can affect the integrity or availability of the 5’ cap.^65–67^ This disruption can interfere with the recruitment of translation initiation factors like eIF4E and impair the assembly of the translation initiation complex. Besides, dysregulated miRNA expression, modification or aberrant alternative splicing of mRNA, can lead to abnormal translation inhibition or activation of specific genes.^42,68–70^ These small RNA molecules target specific mRNAs, such as eIFs, splicing factors and upstream regulators, to affect mRNA secondary structure and modulate their expression. By binding to the 3’ or 5’ UTRs of target mRNAs, miRNAs can inhibit their translations or promote their degradation.

### Regulation of elongation

Deregulation of elongation, the process by which ribosomes moving along the mRNA during protein synthesis, can significantly impact translation efficiency, fidelity, and protein production (Fig. 3a). The regulation of the protein elongation involves modifications of elongation factors (eEFs), errors in aminoacyl-tRNA selection, tRNA mutation, ribosome stalling, modification of ribosome itself, as well as environmental or cellular stress conditions (Fig. 3b).^71,72^

Based on the deregulation mechanisms, such as post modification or mutation, eEFs can disrupt their normal function, leading to defects in elongation.^73,74^ Impaired eEF2 activity can result in reduced ribosome translocation, leading to slower translation rates and potentially affecting protein folding, localization or function.^75^ Deregulation of alternative mRNA splicing encodes abnormal protein isoforms that disturb the biosynthesis of EEF1B2, thus promoting the progression of diseases in eukaryotes.^76^ Furthermore, modifications of mRNA, such as methylation or methyl adenosine, disrupt tRNA selection and decoding in the elongation process.^77,78^ Aminoacyl-tRNAs are selected and delivered to the ribosome based on codon-anticodon recognition. Therefore, deregulation of this process can lead to errors in aminoacyl-tRNA selection and incorporation of incorrect amino acids into the growing polypeptide chain,^79^ resulting in defective or non-functional proteins that contribute to cellular dysfunction or disease. Additionally, mutation of tRNA is reported to cause ribosome stalling leading to premature polypeptide release and neurodegeneration.^80^ Ribosome stalling, a phenomenon where ribosomes become temporarily trapped on the mRNA template, can result in translation errors, premature termination, or formation of abnormal protein structures.^81,82^ This can be caused by various factors, including mRNA secondary structures, codon repeats, rare codons, mRNA damage or limitations in the availability of specific eEFs. Additionally, modifications to ribosomal proteins could modulate the rate of ribosome movement during elongation process.^83–85^ Changes in ribosome speed can influence protein folding, co-translational modifications or interactions with molecular chaperones, which impact the quality and functionality of the synthesized proteins.^61^ Furthermore, various environmental or cellular stress conditions such as oxidative stress, heat shock, nutrient deprivation or viral infection can influence elongation dynamics. Stress-induced deregulation of eEFs, ribosome modifications or the availability of aminoacyl-tRNAs can affect translation elongation and lead to ribosome pausing or changes in the ribosome composition,^86^ thus impacting protein synthesis and cellular adaptation to stress.^87^

Deregulation of elongation can have profound implications on protein synthesis and cellular function. It can result in translation errors, protein misfolding, and alterations in protein abundance or quality, leading to cellular dysfunction, disease pathogenesis or cellular responses to stress. Investigating the mechanisms of elongation deregulation can offer valuable insights into disease processes and potential therapeutic targets.

### Regulation of termination

During the termination process, when the stop codon (UAG, UGA, or UAA) of mRNA enters the ribosomal A-site, the protein releasing factor complex eRF1/eRF3•GTP binds to the A-site instead of activated amino acid tRNA, thereby inducing the termination of protein synthesis (Fig. 3a).^88^ Deregulation of termination, which represents the final stage of protein synthesis, can significant impact translation fidelity and functional proteins production. Abnormal termination occurs due to dysregulated read manner of termination codon and alterations in 3’ UTR of mRNA, ribosome and modifications of termination factors (Fig. 3b).

Normally, termination of translation occurs upon ribosome encountering a stop codon in the mRNA sequence. However, premature termination codons (PTCs) can sometimes be bypassed, and translation continues beyond the intended stop site. This phenomenon, known as PTC readthrough or nonsense suppression, can be induced by various factors, including specific genetic mutations, ribosomal context, or the presence of suppressor tRNAs.^89,90^ PTC readthrough can lead to the synthesis of elongated or abnormal proteins that could potentially alter their function or stability.^90,91^ Additionally, changes in regulatory elements within the 3’ UTR of mRNA can influence translation termination efficiency and lead to deregulated termination, aberrant protein synthesis or altered protein levels.^92^ Ribosome stalling at stop codons is also possible during the process of translation termination.^93^ Stalling can be caused by mRNA secondary structures, cis-acting sequences, or interactions with specific factors. Ribosome stalling at termination codons can result in the accumulation of incomplete polypeptides, triggering cellular quality control mechanisms such as the nonsense-mediated mRNA decay pathway or the ribosome-associated protein quality control pathway. Furthermore, post-translational modifications of termination factors, such as eRFs or ribosomal proteins, can influence their activity or interactions during termination.^94–96^ Alterations in the modification patterns of termination factors may impact their functions and subsequently affect termination efficiency and fidelity. For instance, phosphorylation of eRF1 or eRF3 has been shown to modulate their interactions with the ribosome or other translation factors, thereby impact termination dynamics.^88,97^

Furthermore, deregulation of termination can lead to the production of truncated or abnormal proteins, disrupting protein homeostasis and impacting cellular function. This phenomenon may contribute to disease pathogenesis by generating non-functional or toxic proteins, eliciting cellular stress responses, or interfering normal protein-protein interactions. Therefore, targeting deregulation of termination can provide potential intervention therapeutic for human diseases.

## Human diseases associated with protein translation deregulation

### Neurodegenerative diseases

**Fig. 4:**
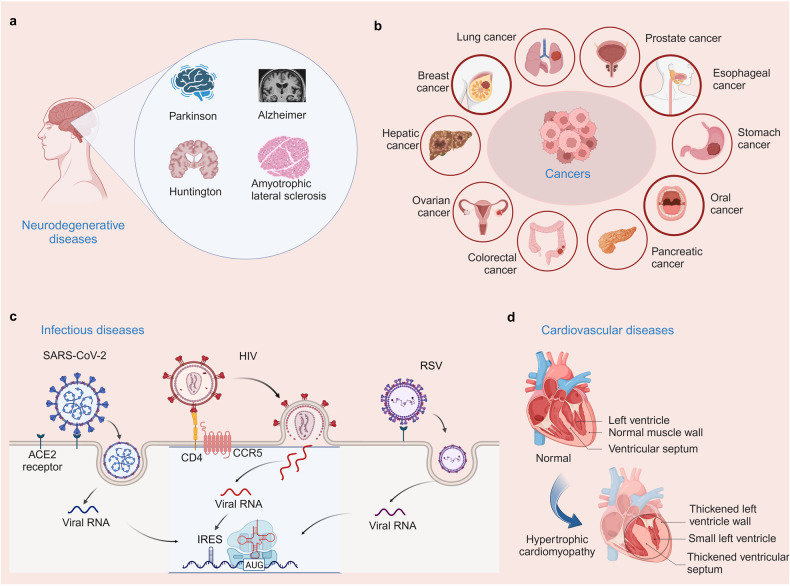
Human diseases associated with protein translation deregulation. **a** Neurodegenerative diseases (Parkinson’s disease, Alzheimer’s disease, Huntington’s disease, and amyotrophic lateral sclerosis) associated with protein translation deregulation. **b** Cancers (including lung cancer, breast cancer, liver cancer, ovarian cancer, colon cancer, pancreatic cancer, oral cancer, stomach cancer, esophageal cancer, and prostate cancer) associated with protein translation deregulation. **c** Infectious diseases (SARS-CoV-2, HIV, RSV) associated with protein translation deregulation. **d** Cardiovascular diseases associated with protein translation deregulation (Hypertrophic cardiomyopathy)

Neurodegenerative diseases encompass a group of debilitating disorders characterized by the progressive loss of neurons in the central nervous system. These diseases, including Parkinson’s disease (PD), amyotrophic lateral sclerosis (ALS), Alzheimer’s disease (AD), and Huntington’s disease (HD), impose a significant burden on global healthcare systems (Fig. 4a).^98^ Despite extensive research efforts, the precise causes and mechanisms underlying these disorders remain elusive. This section provides an overview of the association between protein translation deregulation and neurodegenerative diseases, with a special focus on mRNA, tRNA, translation factors, and ribosome aspects. The development of novel drugs targeting these translation regulators have provided compelling evidence in neurodegenerative diseases.^99–102^ However, most of these disorders progress exhibit rapid progression and currently lack effective treatments capable of halting or reversing disease advancement. Available therapies mainly focus on symptom management or offer only a modest extension to lifespan. This underscores the urgent need to identify compounds that can regulate the affected factors, with the aim of alleviating translational defects or protein accumulation.

Accumulation of evidence showed that chronic dysregulation of cellular processes leads to the gradual build-up of subtle cytotoxic effects, ultimately resulting in premature death of dopaminergic neurons.^103^ Several cellular processes have been extensively studied including protein folding, mitochondrial physiology, membrane physiology, vesicular transport, gene transcription, protein degradation and autophagy.^104^ Recent studies have revealed the involvement of several PD-related proteins in protein translation processes. Abnormal aggregation of α-synaptic nuclear proteins may also occur during the onset of PD and is closely related to protein deregulation. A notable example is eIF4G1, which has been linked to both PD.^105^ and Lewy body dementia.^106^ eIF4G1 is a translation initiation factor that facilitates the recruitment of ribosomes and tRNAs to the 5’ cap structure of mRNA by acting as a scaffold in the eIF4F translation initiation complex.^107^ Additionally, studies have shown the relevance of tRNA enzymes in neurological disorders. Furthermore, a recent study showed an association between a single nucleotide polymorphism in the mitochondrial translation initiation factor 3 gene and PD risk.^108^ Translation factor activity is regulated by signaling pathways like PI3K, mTOR, and MAPKs that modulate general translation factors or factors influencing mRNA transport or stability.^109,110^ For example, the deregulated mTOR pathway in PD controls translation proteins including eIF4E inhibitor 4E-BP, eEF2 kinase, and ribosomal S6K.^111^ ALS is a progressive neurodegenerative disease characterized by loss of motor neurons leading to muscle weakness. Most cases of ALS are sporadic, some similar familial cases exist clinically. When eIF2α is phosphorylated, the global protein synthesis process is attenuated.^112^ The unfolded protein response sensor PERK is activated by high levels of misfolded proteins to phosphorylate and globally inhibit the translation factor eIF2α.^113^ Dysregulated levels of eIF2α and other cellular stress biomarkers have been observed in specimens from ALS patients and disease models.^114^ Modulation of eIF2α phosphorylation or upstream factors has emerged as a potential therapeutic approach. eIF2α undergoes phosphorylation by the guanine nucleotide exchanger eIF2B, leading to inhibition of eIF2B activity by phospho-eIF2α.^115^ eIF2α can be phosphorylated by both PERK and eIF2B. Halliday et al. conducted a screening for safe compounds targeting eIF2α phosphorylation and found that trazodone hydrochloride (a licensed antidepressant) and dibenzoyl methane could reverse p-eIF2α induced translational repression without affecting eIF2α levels. These compounds were able to rescue deficits and provide neuroprotection in vitro as well as in mouse models of prion disease and tauopathy.^99^ As the most prevalent neurodegenerative disorder, AD is characterized aberrant accumulation of misfolded proteins in the endoplasmic reticulum (ER).^116^ In AD, aggregates are composed of the amyloid-β (Aβ) peptide and tau protein, which form extracellular amyloid plaques and intracellular neurofibrillary tangles (NFT), respectively. Although these insoluble aggregates are classical histopathological hallmarks of AD, a substantial body of evidence indicates that soluble oligomeric forms of Aβ (AβOs) and tau (TauOs) are the most neurotoxic forms, inducing brain oxidative stress, mitochondrial damage, deregulation of intracellular signaling pathways, synapse failure and memory deficits.^112^ The key histological findings in AD encompass the accumulation of amyloid precursor protein (APP) and its cleavage into amyloid beta by β-site APP-cleaving enzyme 1 (BACE1). Amyloid beta aggregation forms extracellular plaques and intracellular neurofibrillary tangles. Additionally, the accumulation of tau protein is also gaining attention as a significant feature of AD.^117^ Increased phosphorylation of eIF2α has been observed in postmortem samples from sporadic AD patients and transgenic mouse models.^118^ In 2016, a study reported that Gastrodin suppressed BACE1 expression in the hippocampi of Tg2576 AD mice under oxidative stress by inhibiting the PKR/eIF2α signaling pathway. Furthermore, Gastrodin improved learning and memory while ameliorating oxidative stress in the hippocampi of Tg2576 mice overexpressing the Swedish mutation of the amyloid precursor protein, suggesting it may be a potential candidate of AD treatment.^119^ Genetic or pharmacological inhibition of eIF2α phosphorylation can restore memory and prevent neurodegeneration.^120^ HD is a progressive and fatal neurodegenerative disorder. The pathogenesis of HD is attributed to an expanded CAG trinucleotide repeat in the HTT gene encoding huntingtin protein. This leads to an abnormally elongated polyglutamine tract within the mutant huntingtin protein, which elicits cytotoxicity and neural cell death through both gain-of-function and loss-of-function mechanisms, ultimately leading to the characteristic clinical manifestations of HD.^121^ In terms of protein translation interventions for Huntington’s disease, potential strategies include targeting Huntington’s DNA and RNA as well as promoting protein clearance.^121–123^

Collectively, understanding the intricate relationship between protein translation and neuronal function is essential for the development of effective therapeutic interventions.

### Cancers

In recent years, there has been growing evidence of deregulation in protein translation, which plays a crucial role in the development and progression of various cancers (Fig. 4b). In this section, we provide an overview of protein translation deregulation in different cancers based on the alteration of mRNA, tRNA, translation factors, and ribosome aspects (Table 1).

**Table 1:** Deregulation of RNA, translation factors, ribosome and mTOR pathway aspects in various cancers

Cancer type	Category	Deregulation type	Consequence
Breast cancer	tRNA	tRNA-Leu, tRNA-Tyr	proliferation
	ribosome	RPL15	metastasis
	translation factors	eIF2, eIF4A, eEF2, eEF2K	abnormal expression
	mTOR pathway	S6K, 4E-BP1	metabolism, proliferation
Colorectal cancer	translation factors	eIF4A, eIF3a	proliferation, metastasis, resistance
	tRNA	guanosine hypomodification	ribosome frameshift, oncogenic
	mRNA	M^6^A modifications	abnormal translation encoding
	ribosome	alterations	hyperproliferative and survival factors
	mTOR pathway	ribosomal components upregulated	accelerate protein turnover
Lung cancer	translation factors	eIF4E	abnormal expression
	tRNA	A12172G Mutation	pathogenic
Prostate cancer	translation factors	eIF4A, eIF4E, eEF1A	survival, resistance
	ribosome	PIM1, ribosomal small subunit protein 7	ribosomal stress, advancing tumor progression
Pancreatic cancer	translation factors	eIF5A, eEF1, eIF4E	proliferation, invasion, metastasis
	tRNA	METTL8	tRNA Methylation
	ribosome	ribosome biogenesis	cell proliferation
	mTOR pathway	protein biogenesis decrease	adaptive resistance to KRAS and MEK
Gastric cancer	translation factors	eEF1a, EIF3b, eIF4a	worse outcome alter protein
	tRNA	tRNA-derived fragments	translation, prognostic biomarkers
	ribosome	distribution of RPL22	proliferation
Liver cancer	translation factors	eIFs	closely associated with the development of hepatocellular carcinoma
	mRNA	m6A modification of PDK4	promote tumor growth
	tRNA	modifications	PPARδ translation, cholesterol synthesis
	ribosome	hyperactive ribosome biogenesis	disrupt RPL5 and RPL11 nuclear location, ubiquitylation and degradation of p53
Esophageal cancer	translation factors	EIF3H, eEF1A, eEF2	promote proliferation
	tRNA	N7-methylguanosine tRNA modification	promote tumorigenesis process
	mRNA	microRNA alterations	proliferation, invasion, metastasis
	ribosome	ribosomal protein S6	promote tumor progression

First, depletion of leucyl-tRNA synthetase reduces the abundance of specific leucine tRNAs, thereby affecting leucine codon-dependent translation and promoting tumor formation and proliferation in breast cancer.^124^ Recent studies have demonstrated dysregulation of tRNA expression levels, particularly tRNA-Leu and tRNA-Tyr, in breast cancer.^125,126^ The dysregulation of *RPL15*, a gene that encodes a component of the large ribosomal subunit, significantly affects the protein synthesis process in circulating tumor cells, leading to the accumulation of proliferation and epithelial markers in these cells. Deregulation of *RPL15* expression in ribosomes leads to enhanced translation of cell cycle regulator proteins, thereby promoting tumor metastasis.^127^ Deregulation of translation factors, such as eIF2, eIF4A, eEF2, and eEF2K, is consistently observed in breast cancer.^128–133^ Furthermore, deregulation of the mechanistic target of rapamycin (mTOR) signaling pathway extensively affects protein translation, metabolism, and proliferation in breast cancer.^134,135^ Relatedly, in colorectal cancer, alterations in translation factors, including increased expression levels of eIFs, contribute to enhanced protein synthesis and upregulation of key factors involved in cell proliferation, tumor growth, metastasis, and oxaliplatin resistance.^136,137^ Dysregulated translation also impacts therapy response and development of resistance in colorectal cancer.^138^ Epigenetic loss of tRNA-yW Synthesizing Protein 2 increases guanosine hypomodification of tRNA, inducing ribosome frameshift and leading to the translation of oncogenic genes.^139^ M^6^A modifications of mRNAs result in abnormal translation in colorectal carcinogenesis, affecting various aspects of colorectal cancer.^140,141^ Alterations in rRNA and ribosomal protein biogenesis are closely associated with colorectal cancer cell growth.^142^ In PI3K mutant tumors, ribosomal components are significantly upregulated in an mTOR-dependent manner.^143^

Besides, abnormal expression of translation factors, such as eIF4E, is associated with enhanced cap-dependent translation initiation and increased protein synthesis in non-small cell lung cancer.^144^ Dysregulated translation promotes cell proliferation, survival, and therapy resistance in lung cancer.^145,146^ Mutations in tRNA disrupt its secondary structure and post-transcriptional modifications, affecting protein synthesis in lung cancer.^147^ Relatedly, prostate cancer cells and patient samples exhibit notable changes in translation factors such as eIF4A, eIF4E, and eEF1A.^148–150^ Specifically, eIF4E, which is under the regulation of Heat shock protein 27 (Hsp27), assumes a critical role in enhancing cell survival and fostering resistance to therapies.^121,151,152^ This identifies eIF4E as a promising therapeutic target for advanced prostate cancer. The disruption of translation initiation processes, leading to heightened protein synthesis of components involved in cell growth, survival, and androgen receptor signaling, actively drives the progression of prostate cancer.^153^ Additionally, the aberrant ribosomal biosynthesis of PIM1 and ribosomal small subunit protein 7 leads to ribosomal stress, thereby promoting tumor progression within prostate cancer.^154^ Dysregulation of translation factors, such as eIF5A, eEF1, and eIF4E, contributes to enhanced protein synthesis and upregulation of factors involved in cell proliferation, invasion, and metastasis in pancreatic cancer.^155–159^ Methylation of tRNA by the RNA methyltransferase METTL8 plays an important role in the protein translation process of pancreatic cancer.^160^ Moreover, enhanced ribosome biogenesis contributes to the cell proliferation of RAS-induced or Wnt-dependent pancreatic cancer.^161,162^ Targeting mTORC1/2 has been shown to decrease downstream proteins, thus overcoming adaptive resistance to KRAS and MEK in pancreatic cancer.^163^ Overexpression of eEF1a, eIF3b, and eIF4a indicates a worse outcome for cancer patients in gastric cancer.^164–166^ Dysregulation of tRNA-derived fragments (tRFs) can displace RNA binding proteins and alter protein translation, making them potential diagnostic and prognostic biomarkers for gastric cancer.^167^ The distribution of L22 ribosomal protein in the nucleus and cytoplasm has been positively associated with gastric cancer proliferation.^168^ Additionally, in liver cancer, modification and abnormal expression of eIFs are closely associated with the development of hepatocellular carcinoma induced by chronic hepatitis C or chronic hepatitis B.^169^ The m^6^A modification in the 5’ UTR of PDK4 promotes hepatoma tumor growth by binding with eEF2.^170^ Furthermore, deregulation of tRNA modifications can affect PPARδ translation and trigger cholesterol synthesis in the liver tumorigenesis.^171^ Overexpression of small nucleolar RNA H/ACA box leads to hyperactive ribosome biogenesis and disrupts the nuclear location of ribosomal proteins RPL5 and RPL11, resulting in the ubiquitylation and degradation of p53.^172^ Translation factors such as eIF3H, eEF1A, and eEF2 have been reported to promote proliferation of esophageal cancer cells.^173–175^ Deregulation of N7-methylguanosine tRNA modification (m7G) has been found to be essential for the tumorigenesis process of esophageal cancer.^176^ Alterations in microRNA have been observed to lead to aberrant expression and translation of mRNA, thereby promoting cancer cell proliferation, invasion, and metastasis.^177–179^ Phosphorylation of ribosomal protein S6 has also been found to be closely related to the progression of esophageal cancer.^180^ In addition to the above-mentioned cancers, protein translation deregulation is also involved in the tumorigenesis process of ovarian cancer and oral cancer.^163,181^

Overall, elucidating the mechanisms of protein translation deregulation in cancer is crucial for the development of targeted therapies. Strategies aimed at translation factors, upstream modification targets of mRNA, tRNA, and ribosomes, or global translational control exhibit promising in impeding tumor growth, overcoming therapy resistance, and improving cancer treatment outcomes.

### Infectious diseases

In infectious diseases, protein translation deregulation is a common characteristic, and is observed playing a crucial role in their pathogenesis (Fig. 4c). This dysregulation involves various aspects, such as alterations in translation initiation,^182^ changes in translation elongation,^183^ and the involvement of regulatory factors and signaling pathways, all of which contribute to the abnormal synthesis of proteins. Dysregulation of translation initiation can result in the preferential synthesis of viral proteins, enabling the pathogen to evade host immune surveillance.^184^ Similarly, alterations in translation elongation can impact the synthesis of host defense proteins, compromising immune responses. Additionally, the involvement of regulatory factors and signaling pathways further modulates protein translation, influencing disease outcomes. Therefore, targeting translation initiation and eEFs, regulatory factors, or signaling pathways involved in protein translation deregulation may offer potential therapeutic strategies.

Viral reproduction is contingent on viral protein synthesis that relies on the host ribosomes. As such, viruses have evolved remarkable strategies to hijack the host translational apparatus in order to favor viral protein production and to interfere with cellular innate defenses.^185^ COVID-19 is a viral inflammatory disease primarily affecting the lungs. SARS-CoV-2 replication in the lungs induces inflammatory and immune responses, leading to respiratory issues, systemic effects, and lung damage.^186^ SARS-CoV-2 viral proteins interact with the host translation machinery. Inhibitors targeting translation have shown potent antiviral effects against SARS-CoV-2. Plitidepsin, for example, has been found to exhibit antiviral activity against SARS-CoV-2 by inhibiting eEF1A.^183^ Another study by Sidharth Jain et al. discovered that C16 is a dual inhibitor of EIF2AK and SARS-CoV-2N, exhibiting strong antiviral activity.^187^ Viruses evade the innate immune response by suppressing the production or activity of cytokines such as type I interferons (IFNs). The SARS-CoV-2 NSP2 protein impedes Ifnb1 mRNA translation by hijacking the GIGYF2/4EHP complex. This evades the innate immune response and promotes viral replication.^188^ miRNAs and tRFs have emerged as important elements in controlling viral replication and host responses. Studies have shown that certain tRFs are highly over-expressed in liver biopsies from patients with chronic hepatitis and hepatocellular carcinoma.^189^ Furthermore, in the case of human respiratory syncytial virus (RSV), which commonly causes bronchiolitis and pneumonia in infants, RSV infection leads to the abundant production of 30-nt tRFs known as tRNA-derived stress-induced RNAs (tiRNAs).^190^ These tiRNAs have been found to bind to apolipoprotein E receptor 2 (APOER2) and suppress its expression, thereby promoting RSV replication.^191^ Similarly, tRF3 has been shown to target the HIV-1 virus through RNA interference, and its cellular prevalence is positively correlated with HIV proliferation.^192^ Modulating translation initiation or elongation can disrupt viral protein synthesis, restore host defense protein synthesis, and enhance immune responses. For example, the discovery of oxazole-benzenesulfonamides has shown promise in inhibiting HIV-1 replication by disrupting the RT-eEF1A interaction, offering potential anti-HIV drugs.^193^ Certain pathogens can exploit the host cell’s unfolded protein response (UPR) pathway to exert beneficial effects. For instance, Plasmodium and Newcastle disease virus activate UPR to promote host cell apoptosis, facilitating pathogen release; hepatitis C virus manipulates UPR signaling to reprogram host cell metabolism, optimizing the intracellular environment for its replication.^182^ Meanwhile, UPR activation can also increase inflammatory cytokine expression and apoptosis in host cells, eliciting immune responses. Therefore, effector molecules like YopJ in Plasmodium and NS5A in hepatitis C virus can inhibit UPR, helping pathogens evade immune surveillance.^194^ The pathogen-host interplay at the translational level impacts disease outcomes. Elucidating these complex interactions hold promise for treating infections.

### Cardiovascular diseases

Emerging evidence suggests that protein translation deregulation plays a critical role in the development and progression of CVDs (Fig. 4d).^195^ Although specific CVDs and their associated cardiometabolic abnormalities have distinct pathophysiological and clinical manifestations, they often share common traits, including disruption of proteostasis resulting in accumulation of unfolded or misfolded proteins in the ER.^185^ Preliminary findings indicate that protein translation deregulation contributes to the pathogenesis of CVDs through multiple mechanisms. Cardiac gene expression is extensively controlled at the translational level in a process-specific manner. Analysis of 80 human hearts uncovered extensive translational regulation of cardiac gene expression, including inefficient translation termination, leading to identification of many novel microproteins with diverse cellular functions.^195^ Dysregulated translation leads to altered expression of key proteins involved in cardiac contractility, endothelial function, and vascular remodeling. Moreover, the deregulation affects the synthesis of proteins implicated in oxidative stress, inflammation, and lipid metabolism, all of which are implicated in CVDs. Additionally, aberrant translation can disrupt protein quality control mechanisms, resulting in the accumulation of misfolded proteins and cellular dysfunction.^196^ Recent research has shown that the binding of TIP30 to the eEF1A prevents its interaction with its essential co-factor eEF1B2, thereby inhibiting translational elongation. This suggests that TIP30 could be therapeutically targeted to counteract cardiac hypertrophy.^197^ Stimulating PI3K and inhibiting PTEN, which degrades inositol 3, 4, 5-trisphosphate, increases cardiac cell size. This is also seen with a constitutively active Akt mutant, along with increased phosphorylation of 4EBP1 and S6K1.^198^ Administration of rapamycin attenuates the cardiac hypertrophy resulting from Akt expression, further demonstrating the involvement of mTOR as the mediating effector.^199^ Additionally, understanding the molecular mechanisms underlying protein translation deregulation in CVDs may facilitate the development of novel diagnostic biomarkers and personalized treatment strategies. Further research is warranted to elucidate the precise mechanisms involved and explore potential therapeutic interventions for CVDs.

## Techniques used to study protein translation deregulation

**Fig. 5:**
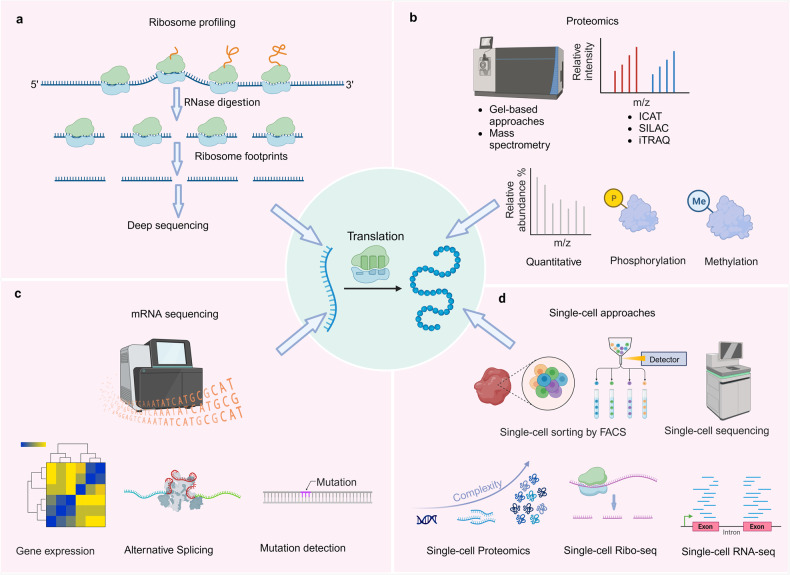
Techniques used to study protein translation deregulation. **a** Ribosome profiling, a technique used to study protein translation deregulation. **b** Mass spectrometry-based proteomics, including protein quantification, phosphorylation quantification, and methylation quantification, used to study protein translation deregulation. **c** mRNA sequencing, including gene expression, alternative splicing, and mutation detection, used to study protein translation deregulation. **d** Single-cell approaches, including single-cell proteomics, single-cell ribosome profiling, and single-cell transcriptomics, used to study protein translation deregulation

In the course of research, protein translation deregulation is inseparable from the utilization of various techniques that provide valuable insights into the molecular basis of this process. Ribosome profiling,^200^ mass spectrometry-based proteomics.^201^ and single-cell translation profiling.^202^ each possess unique advantages and limitations. Integrating these techniques enable a comprehensive understanding of protein translation deregulation (Fig. 5). Continued advancements in these techniques, along with their integration with other omics approaches, will undoubtedly contribute to further unraveling the intricate mechanisms underlying the deregulation.

### Ribosome profiling

Ribosome profiling is a technique that involves sequencing ribosome footprints, which are short fragments of mRNA (Fig. 5a).^203^ These footprints are protected from nuclease digestion because they are physically enclosed by the ribosome. These footprints are then converted into a library of DNA fragments for analysis by next-generation sequencing.^204^ Ribosome profiling enables precise measurement of translation levels, overcoming limitations of traditional methods.^205^ By capturing ribosome-protected mRNA fragments, ribosome profiling provides valuable insights into translation initiation, elongation, and termination. This innovative approach allows for meticulous monitoring of each stage of protein synthesis in vivo and facilitates quantification of protein synthesis rates across the entire proteome. Moreover, ribosome profiling has provided invaluable insights into the mechanism of action of various anticancer drugs that specifically target the translational apparatus.^206^ For instance, rocaglates selectively kill cancer cells by targeting eIF4A.^207^ Ribosome profiling has revealed that these drugs specifically inhibit translation of certain mRNAs.^208^ Unlike conventional eIF4A inhibitors, rocaglates clamp eIF4A onto polypurine RNA sequences, acting as roadblocks to translation initiation.^209^ By employing this technique, researchers have been able to precisely examine the effects of these drugs on protein synthesis within cancer cells. Ribosome profiling has enabled the observation changes in translation dynamics and identification of alterations in the ribosome occupancy on specific mRNA transcripts upon drug treatment. This information has shed light on the specific targets and pathways affected by these anticancer drugs, allowing for a better understanding of their mode of action. Moreover, they facilitate the annotation of the protein coding potential of genomes, the examination of localized protein synthesis, and the exploration of co-translational folding and targeting phenomena. The wealth of data generated by ribosome profiling possesses an unparalleled quantitative nature, presenting an unprecedented opportunity to investigate and model intricate cellular processes.^210^

### Mass spectrometry (MS)-based proteomics

In recent years, mass spectrometry proteomics has emerged as a powerful tool for investigating dynamic changes in protein translation and identifying key players (Fig. 5b). By pulse-labeling nascent peptide chains with heavy amino acid isotopes (SILAC) or click-reactive amino acids/puromycin, mass spectrometry approaches can assess protein dynamics like degradation and synthesis. Quantitative proteomics with mass spectrometry can compare overall protein levels between healthy and diseased cells/tissues, revealing which proteins are over/under produced due to defects in translation.^211^ Chemical labeling and pulse-chase assays tracked by mass spectrometry can monitor the kinetics of protein synthesis and degradation, uncovering abnormalities in translation disorders.^212^ This sensitive method of proteomics also reveals the role of eIF2 pathways in regulating translation for cellular survival under stress.^213^ Additionally, proteomics analysis enhances our understanding of the drug reaction of eIF4A targeting compound zotatifin.^214^ Moreover, integrating MS proteomics with other omics approaches is crucial for gaining a comprehensive understanding of protein translation dysregulation. Through the identification and quantification of differentially translated proteins, as well as the characterization of translational regulatory mechanisms, mass spectrometry-based proteomics provides valuable insights into the molecular basis of protein translation deregulation.^215,216^ Integration with other omics data further enhances our understanding of this complex process. Continued advancements in mass spectrometry technology and data analysis methods will undoubtedly contribute to further unraveling the intricate mechanisms underlying the deregulation.

### mRNA sequencing

mRNA sequencing is a powerful technique for profiling the transcriptome and has emerged as a valuable tool for investigating protein translation deregulation (Fig. 5c). Moreover, we highlight the importance of integrating mRNA sequencing with other omics approaches to gain a comprehensive understanding of translation deregulation.^217^ Bioinformatic analysis of mRNA sequences can identify motifs or structures that result in ribosome stalling during faulty translation. Through mRNA sequencing, mutations in the Kozak sequence, which plays a role in protein translation, can be identified.^218^ Additionally, mRNA sequencing enables identification of the 5’ UTRs of eukaryotic mRNAs involved in eukaryotic translation regulation.^219^ Investigation into the molecular basis of protein translation deregulation necessitates a thorough comprehension of the transcriptome. By enabling high sensitivity and accuracy in profiling the entire transcriptome, mRNA sequencing has revolutionized the field of transcriptomics. It offers valuable insights into the molecular basis of the deregulated protein through identification of differentially expressed genes, detection of alternative splicing events, characterization of translational efficiency, and discovery of translational regulatory elements. Integration with other omics approaches further enhances our understanding of this complex process. Ongoing improvements in mRNA sequencing technology and data analysis methods will undoubtedly contribute to further unraveling the intricate mechanisms underlying protein translation deregulation.

### Single-cell approaches

Traditional bulk RNA sequencing methods provide an average measurement of gene expression across a population of cells, masking the heterogeneity that exists within individual cells. Single-cell approaches have emerged as powerful tools for dissecting the intricate dynamics of protein translation at the single-cell level (Fig. 5d). Single-cell ribosome sequencing (scRibo-seq) combines nuclease foot-printing, small-RNA library construction and size enrichment to measure translation dynamics in individual cells.^217^ Single-cell proteomics has provided valuable information about protein translation dynamics during cellular differentiation.^220^ Single-cell RNA-seq can define cell-to-cell heterogeneity in gene expression, revealing how translation defects may arise in subpopulations of cells. Single-cell ribosome profiling quantifies translation at the codon level in individual cells, uncovering cell-specific translational dysregulation.^221^ The use of single-cell approaches to study protein translation deregulation has revolutionized our understanding of cellular heterogeneity and dynamics. These techniques have the potential to uncover novel therapeutic targets and biomarkers for various diseases. However, several challenges need to be addressed, including the limited sensitivity and throughput of current methods, as well as the integration of multi-omics data. Future advancements in single-cell technologies, such as the development of high-throughput single-cell proteomics methods and the integration of transcriptomic and proteomic data will further enhance our understanding of translation deregulation.

## Therapeutic strategies targeting protein translation deregulation

### Targeting translation initiation

Extensive research has shown that eIFs play a crucial role in modulating translation and are closely associated with various diseases. In particular, targeting eIFs has emerged as a promising strategy for therapeutic interventions against cancer, as supported by current studies.^222^

Recent studies have revealed that different subunits of eIF3 exhibit varying expression patterns in tumors and are believed to have either oncogenic or tumor suppressor functions.^223^ The eIF3a subunit has been implicated in various cellular processes including the cell cycle, DNA synthesis/repair, differentiation, fibrosis, and tumorigenesis.^224^ Increased expression of eIF3a has been associated with the maintenance of malignant phenotypes in numerous types of tumor.^222^ eIF3a is highly expressed in lung cancer tissues and influences lung cancer patient response to platinum chemotherapy by regulating DNA repair protein expression.^225^ Additionally, the presence of anti-eIF3a autoantibodies has been identified as a potential diagnostic biomarker for hepatocellular carcinoma.^226^ eIF3a regulates HIF1α-dependent glycolytic metabolism in hepatocellular carcinoma cells through translational regulation, and is involved in tumor metabolism.^223^ Given its significant role in carcinogenesis, eIF3a has emerged as a promising therapeutic target for inhibiting tumor proliferation. The small molecule NCE22 has demonstrated cytotoxicity against tumor cells in vitro by acting as an inhibitor of eIF3a.^227^ Ongoing studies are currently investigating the potential clinical applications and benefits of eIF3 subunits for patients. EIF3b expression has been found to be associated with the prognosis of bladder and prostate cancer, suggesting that targeting eIF3b could open up new possibilities for cancer therapeutics.^228^ The role of eIF3e in cancer is controversial. Elevated levels of eIF3e correlate with prolonged progression-free survival in tamoxifen-treated breast cancer patients. Additionally, mutations in eIF3e contribute to the malignant phenotype of mammary epithelial cells.^229^ Conversely, low eIF3m expression is an unfavorable indicator, associating with reduced overall, relapse-free, and post-progression survival in breast cancer and colon adenocarcinoma patients.^230^ EIF3h is upregulated in various cancers, including breast cancer, hepatocellular carcinoma, lung cancer, and colon cancer.^231^ In vitro studies have demonstrated that the beta-carboline derived from harmine, CM16, targets eIF3h and exhibits anti-cancer effects.^232^ Conversely, eIF3f acts as a tumor suppressor in cancers like melanoma and pancreatic cancer. Overexpressing eIF3f inhibits cell proliferation and induces apoptosis.^233^ In lymphoma models, inhibiting eIF4A and translation can restore chemosensitivity to doxorubicin.^234^ eIF4F inhibition may also decrease PD-L1 expression and stimulate anti-tumor immunity in melanoma.^235^ eIF4A inhibition with silvestrol suppresses Sin1 translation and attenuates invasion in colon carcinoma.^236^

In addition, the mRNA 5’ cap-binding protein, eIF4E, plays a crucial role in facilitating mRNA translation and promoting the translation of oncogenic mRNAs, including cyclin D3, vascular endothelial growth factor, and Mcl-1.^237^ Furthermore, increased eIF4E activity reduces tumor cell sensitivity to BRAF inhibitors.^238^ The eIF4F complex also associates with resistance to BRAF and MEK inhibitors in BRAF V600-mutant melanoma, colon, and thyroid cancer cells.^239^ Small molecule inhibitors targeting the eIF4E-eIF4G interaction domain, like Quabain and Perillyl alcohol, have been investigated.^153^ These eIF4E inhibitors mimic the eIF4G or 4E-BP1 domain to bind eIF4E. A high-throughput approach identified the eIF4F inhibitor 4EGI-1, which disrupts eIF4F and inhibits cap-dependent translation. 4EGI-1 has shown anti-tumor activity by inhibiting proliferation and inducing apoptosis in Jurkat T-ALL and A549 cancer cell lines in vitro.^240^ Moreover, Ribavirin, an eIF4E inhibitor, has shown striking improvements as monotherapy in an 11-patient clinical trial for acute myeloid leukemia.^241^

### Targeting translation elongation

Elongation involves tRNA entering, peptide bond formation, and ribosome translocation along mRNA.^71^ In mammals, eEF1A, similar to bacterial EF-Tu, binds aminoacyl-tRNA in a GTP-dependent manner and guides it to the ribosomal A-site. GTP hydrolysis by eEF1A occurs upon codon recognition between mRNA and tRNA. eEF1A-GDP is recycled to eEF1A-GTP with help from the eEF1B (α, δ, γ subunits) guanine exchange factor.^219^ Understanding the intricacies of translation elongation are essential for unraveling the mechanisms of protein synthesis.

In the context of cancer, inhibiting translation elongation can selectively impact rapidly dividing cancer cells, leading to growth arrest or apoptosis.^242^ In neurodegenerative disorders, enhancing translation elongation can facilitate the production of functional proteins, thereby mitigating disease progression.^243^ Moreover, modulation of translation elongation can be utilized to combat viral infections by disrupting the synthesis machinery of viral proteins.^244^ These findings underscore the broad therapeutic implications of targeting translation elongation. This tightly regulated process is orchestrated by a complex interplay of various factors, including ribosomes, tRNAs, and eEFs. Current elongation inhibitors primarily target the elongation factors eEF1 and eEF2, as well as the ribosomal A-, P- or E-sites.^245^ Mutations in mitochondrial elongation factors EF-Tumt, EF-Tsmt, EF-Gmt and their cytosolic counterparts eEF1A, eEF1B, eEF2 associate with human diseases affecting the central nervous system.^246^

### Targeting translation termination

Targeting strategies for translation termination initiation holds great promise for therapeutic intervention. In eukaryotes, eRF1 and eRF3 are the primary RFs responsible for recognizing stop codons and promoting peptide release.^247^ Dysregulation of these factors can result in premature termination or readthrough of stop codons, leading to abnormal protein synthesis. Small molecules that selectively target translation termination initiation factors have shown promise as potential therapeutics. For instance, compounds that enhance eRF1 binding to stop codons can promote efficient termination, preventing premature termination or readthrough.^88^ Conversely, inhibitors that disrupt eRF1 or eRF3 function can be employed to modulate translation termination initiation and restore protein synthesis. PF846 inhibits translation termination by slowing elongation and trapping the nascent chain on the ribosome, suppressing the catalytic activity of the peptidyl transferase center (PTC) that is normally stimulated by eukaryotic release factor 1 (eRF1).^248^ SRI-37240 and SRI-41315 promote cystic fibrosis transmembrane conductance regulator (CFTR) nonsense mutation suppression by prolonging translational pause and reducing eRF1 abundance, synergistic with aminoglycosides.^249^

### Preclinical and clinical studies of translation-targeted therapies

Translation-targeted strategies have garnered significant attention due to their ability to modulate protein synthesis and their potential for precision medicine.^250^ Preclinical studies have demonstrated their efficacy in various disease models, including cancer, neurodegenerative disorders, and CVDs.^36^

By specifically targeting the translational machinery, these therapies offer a unique and targeted treatment approach. In the field of cancer research, translation-targeted therapies have shown promise in inhibiting tumor growth.^244^ Similarly, in neurodegenerative disorders, these therapies have been shown to modulate protein synthesis and alleviate disease pathology.^99^ Additionally, translation-targeted therapies have exhibited potential in CVDs by targeting specific proteins involved in cardiac remodeling. Clinical trials evaluating these therapies have demonstrated favorable safety profiles, tolerability, and preliminary efficacy. As an illustration, clinical trial for cancer treatment showed a translation-targeted therapy’s safety and preliminary evidence of antitumor activity (Table 2).^251^ While translation-targeted therapies offer a promising avenue for precision medicine by directly modulating protein synthesis, several challenges need to be addressed for successful translation into clinical practice. These challenges include off-target effects, drug resistance, and delivery strategies, which require further investigation. Additionally, identifying optimal therapeutic targets and developing personalized treatment approaches are crucial for maximizing the efficacy of translation-targeted therapies. Preclinical and clinical studies have provided valuable insights into the development and application of translation-targeted therapies. Further research is warranted to optimize therapeutic strategies, overcome challenges, and improve patient outcomes.

**Table 2:** Small molecule inhibitors targeting translation regulators

Target	Agent	Clinical stage	Reference
eIF4E	Ribavarin, LY2275796 Homoharringtonine	Phase II (NCT00559091) Phase I (NCT00903708) Phase III (NCT05457361)	^252–254^
eIF4E-eIF4G interaction	4EGI-1, 4E1RCat, Quabain	Preclinical	^153,255^
eIF4A	Pateamine A, Rocaglates, Silvestrol	Preclinical	^234,256,257^
eIF3a	Mimosine	Preclinical	^258^
eIF2a	Salubrinal, Troglitazone	Preclinical	^259^
eIF3h	CM16	Preclinical	^232^
mTOR	Rapamycin, Everolimus, Temsirolimus	Approved by FDA	^260^
eEF2K	NH125, rottlerin	Preclinical	^261,262^

## Conclusion and future perspectives

Dysregulation of translation can lead to abnormal protein expression, altered protein isoforms, and disrupted cellular functions, which are often associated with various human diseases. Here, we summarize the deregulation of protein translation based on the alterations in tRNA, mRNA, ribosome and related translation factors. This provides new insights for classifying the deregulations of protein translation in human diseases, such as neurodegenerative diseases, cancer, infectious diseases and CVDs. We also discuss the challenges regarding candidate targets and their related inhibitors that have been evaluated in pre-clinical study. With the advancements in techniques used to study protein translation, mutations, modifications or other disorders in translation elements will be identified in the future researches. However, effective and actionable biomarker targeting translation process and its related inhibitors are still lacking in the clinical for human disease. Continued efforts are needed to discover novel targets and biomarkers focusing on alterations of translation factors, tRNA, mRNA and ribosomes implicated in disease pathogenesis. In addition, further research on rare genetic diseases caused by mutations in translational factors or ribosomal proteins can deepen our understanding of translation mechanisms and offer potential therapeutic approaches for more prevalent diseases. Furthermore, targeting patient-specific alterations in translation which are detected by integrating genomic, transcriptomic, and proteomic data can improve treatment outcomes by tailoring treatments to individual patients based on their specific translation deregulation profiles and holds promise for precision medicine and personalized treatments.

Translation deregulation, with its intricate molecular interactions, reveals the inner mechanics of cellular processes and carries significant consequences for health and disease. The ability to modulate this mechanism, specifically targeting stages such as initiation, elongation, or termination, facilitates the emergence of previously unattainable precision medicine approaches. This research calls for the creation of an advanced approach to engage with cellular processes, facilitating the development of individualized treatment strategies and deepening our understanding of molecular biology. In this domain, the complexities involved in the cellular protein synthesis process pose both a challenge and an opportunity, paving the way for innovative therapeutic methods and a future where diseases are confronted at their cellular foundation.

Overall, a collaborative, multidisciplinary approach that integrates genomics, proteomics, bioinformatics, and functional studies will be essential for advancing our understanding of protein translation deregulation and its implications in human disease. By addressing these future research directions, we can pave a way towards more effective and personalized treatments, ultimately making significant progress in combating various diseases and improving global health outcomes.
